# An Incidental Finding of Concomitant Medullary Thyroid Carcinoma and Papillary Thyroid Carcinoma: A Case Report

**DOI:** 10.7759/cureus.73202

**Published:** 2024-11-07

**Authors:** Mohammad Alandejani, Suhaib Radi, Ammar Tashkandi, Jehad Alzahrani

**Affiliations:** 1 Department of Internal Medicine, Division of Endocrinology, Ministry of National Guard-Health Affairs, Jeddah, SAU; 2 College of Medicine, King Saud Bin Abdulaziz University for Health Sciences, Jeddah, SAU; 3 Department of Medicine, King Abdullah International Medical Research Center, Jeddah, SAU; 4 College of Medicine, Northern Border University, Arar, SAU

**Keywords:** fine-needle aspiration, medullary thyroid carcinoma, papillary thyroid carcinoma, thyroid cancer, thyroid nodule

## Abstract

Concomitant medullary thyroid carcinoma and papillary thyroid carcinoma in the thyroid gland are rare; however, the incidence is increasing. Here, we present the case of a 77-year-old male who was incidentally found to have thyroid nodules after a road traffic accident. Fine-needle aspiration revealed two inconclusive Bethesda classifications: follicular neoplasm and unsatisfactory. The diagnosis of concomitant medullary thyroid carcinoma and papillary thyroid carcinoma was later confirmed by histopathology results after total thyroidectomy. The selected treatment was a total thyroidectomy in addition to radioactive iodine later on due to complications of invasion of the trachea. This case focuses on the possibility of having multiple types of carcinoma in a single thyroid gland.

## Introduction

The most common endocrine malignancy and the fastest-increasing cancer worldwide is thyroid cancer [1]. Four primary types of thyroid carcinomas constitute the majority of cases. The most common is papillary thyroid carcinoma (PTC), which constitutes approximately 80% of all thyroid cancers, followed by follicular thyroid carcinoma (FTC), which accounts for approximately 10-15% of thyroid cancers, medullary thyroid carcinoma (MTC), which accounts for only approximately 3-5%, and anaplastic thyroid carcinoma (ATC), which accounts for 1-2% of cases [2].

Concomitant MTC‐PTC is a rare phenomenon. The incidence of the two forms varies, and over the past two decades, the incidence of MTC‐PTC has increased from 2.7% to 12.3% of all MTCs [3]. A thyroid nodule or neck mass is the initial sign, and flushing or diarrhea accompanied by high calcitonin levels may occur [4]. We present a case where none of these symptoms were reported. Our patient presented to the emergency room (ER) after a road traffic accident (RTA) and was diagnosed only upon testing.

## Case presentation

**Figure 1:**
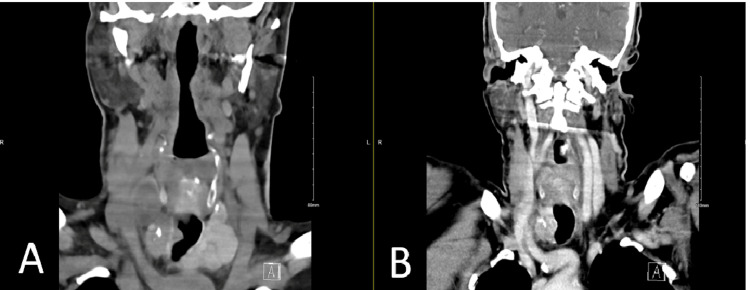
CT scan showing tracheal invasion. (A) CT scan showing preoperative right tracheal invasion. (B) CT scan showing the remaining postoperative right tracheal invasion.

A 77-year-old male known to have diabetes mellitus and hypertension presented to the emergency department after an RTA. At the time, he was complaining only of chest pain and shortness of breath (SOB) that began after the RTA. The emergency and trauma team performed a full assessment, including brain, neck, chest, abdomen, and pelvis computed tomography (CT) scans. After the evaluation, the chest pain and SOB were found to be attributable mainly to non-displaced lift second, third, and fourth rib fractures, as well as sternal hematoma. Further, during an extensive assessment, there was an incidental discovery in the neck CT of bilateral thyroid nodules with invasion of the right side of the trachea (Figure 1).

**Figure 2:**
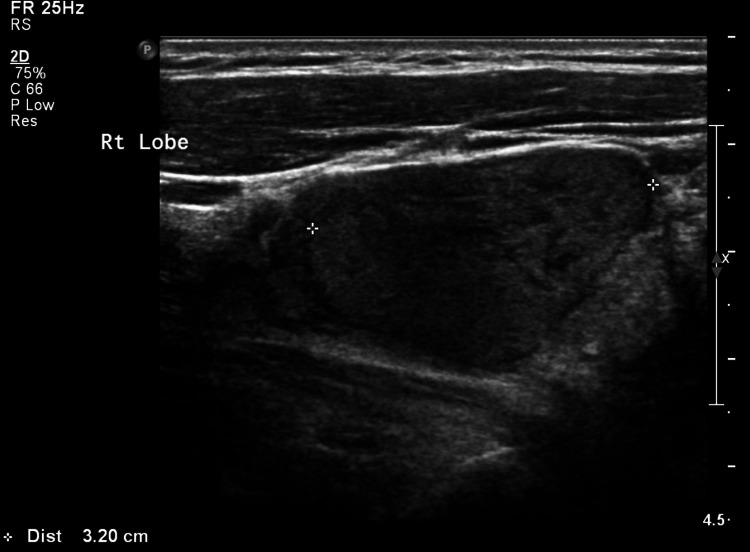
Largest thyroid nodule. Right thyroid ultrasound image showing the largest nodule measuring 3.2 cm.

After two days of admission during which the patient was stabilized, the focus shifted to the incidental findings of the bilateral thyroid nodules and one’s invasion of the right side of the trachea. A thyroid ultrasound (US) was performed to obtain more accurate imaging. The US showed five thyroid nodules, three on the right measuring 3.2 cm, 2.6 cm, and 0.6 cm TIRAD-4, and two on the left measuring 2 cm and 0.6 cm TIRAD-3 (Figure 2). Immediately thereafter, a bilateral thyroid fine-needle aspiration (FNA) was performed on the most prominent nodules on both sides; however, the results were inconclusive. There was a follicular neoplasm in the right FNA, and the evaluation of the left FNA was unsatisfactory. After excluding any severe or critical illness, the patient was discharged on the fifth day. Later, during a follow-up with the patient’s otolaryngologist, the decision was made to perform a total thyroidectomy (TTx).

The TTx was conducted four months after the thyroid FNA. During the TTx, the surgeon noticed that the right lower thyroid lobe had invaded the trachea. However, as there was no tissue diagnosis and staging at that time, the surgeon decided not to remove the tracheal lesion. After the specimens were sent to pathology, the thyroid resection showed a right upper MTC measuring 0.7 cm, a right lower PTC measuring 2.5 cm, and a left lobe PTC measuring 0.9 cm. None of the lesions showed any angioinvasion, lymphatic invasion, or necrosis. After the diagnosis of thyroid cancer MTC and PTC was confirmed, the patient was referred to the endocrine department.

Clinically, the patient had complications only of recurrent nerve injury post-TTx with hoarseness of voice. Upon further assessment, the patient’s laboratory levels were as follows: calcitonin 2 pg/mL, carcinoembryonic antigen (CEA) 10 ng/mL, thyroglobulin (TG) 19 ng/mL, thyroglobulin antibody 5 ng/mL, thyroid-stimulating hormone (TSH) 0.1 mIU/L, parathyroid hormone 40 pg/mL, metanephrine 38 pg/mL, and normetanephrine 87 pg/mL. Genetic testing associated with thyroid cancer was performed and did not reveal any of the known mutations, such as *RET* gene mutation for MEN2A, MEN2B, or familial MTC. Because the patient had a high risk of PTC with invasion of the trachea, we recommended a revision of TTx, but the patient refused any further surgery. Instead, he received diagnostic radioactive iodine (RAI) with a 131-whole body scan at a dose of 3 mCi, which resulted in intense uptake in the right thyroid tissue.

**Figure 3:**
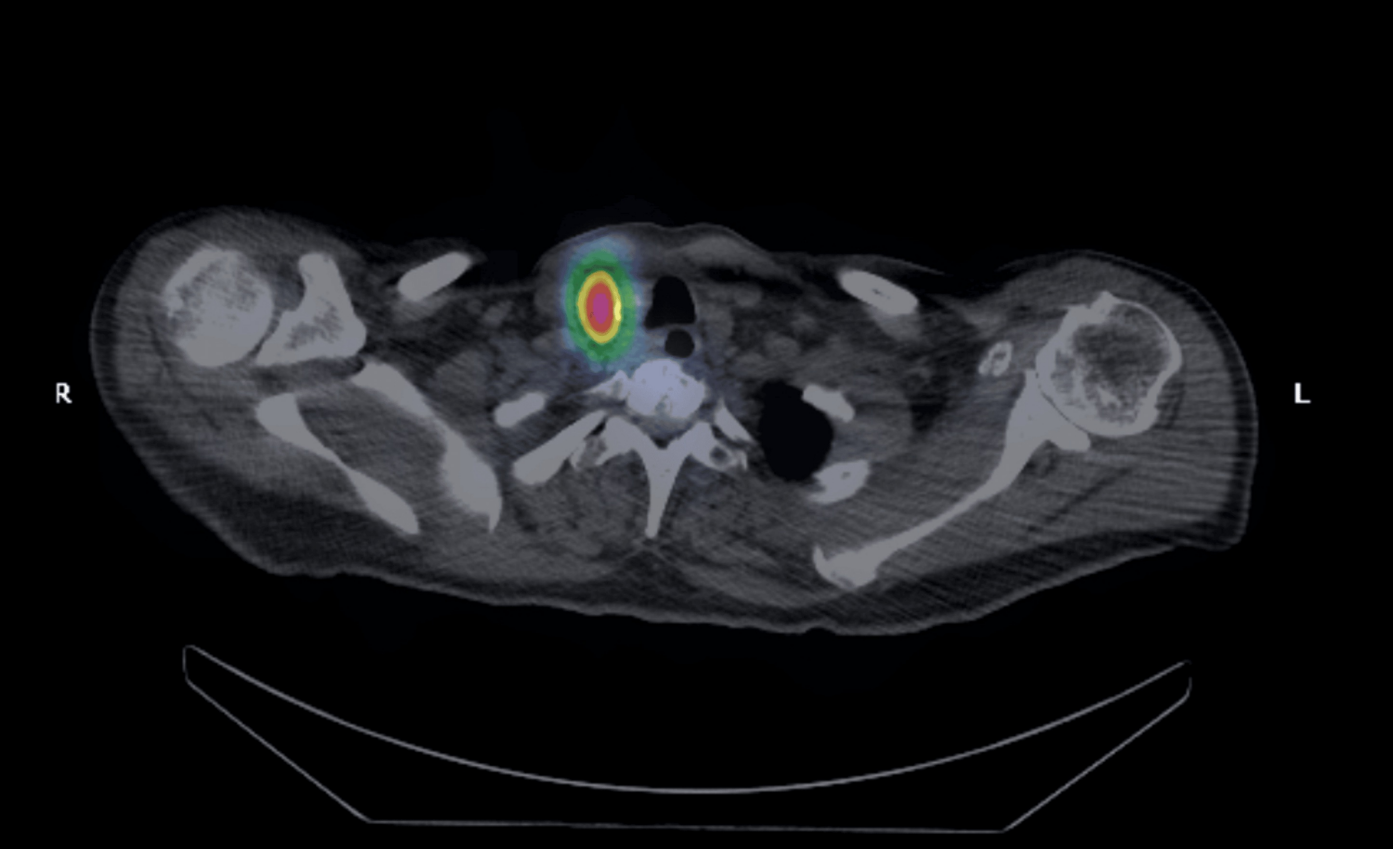
131-Whole body scan at a dose of 150 mCi. Intense uptake in the right thyroid bed is colored red centrally and represents residual thyroid disease.

**Figure 4:**
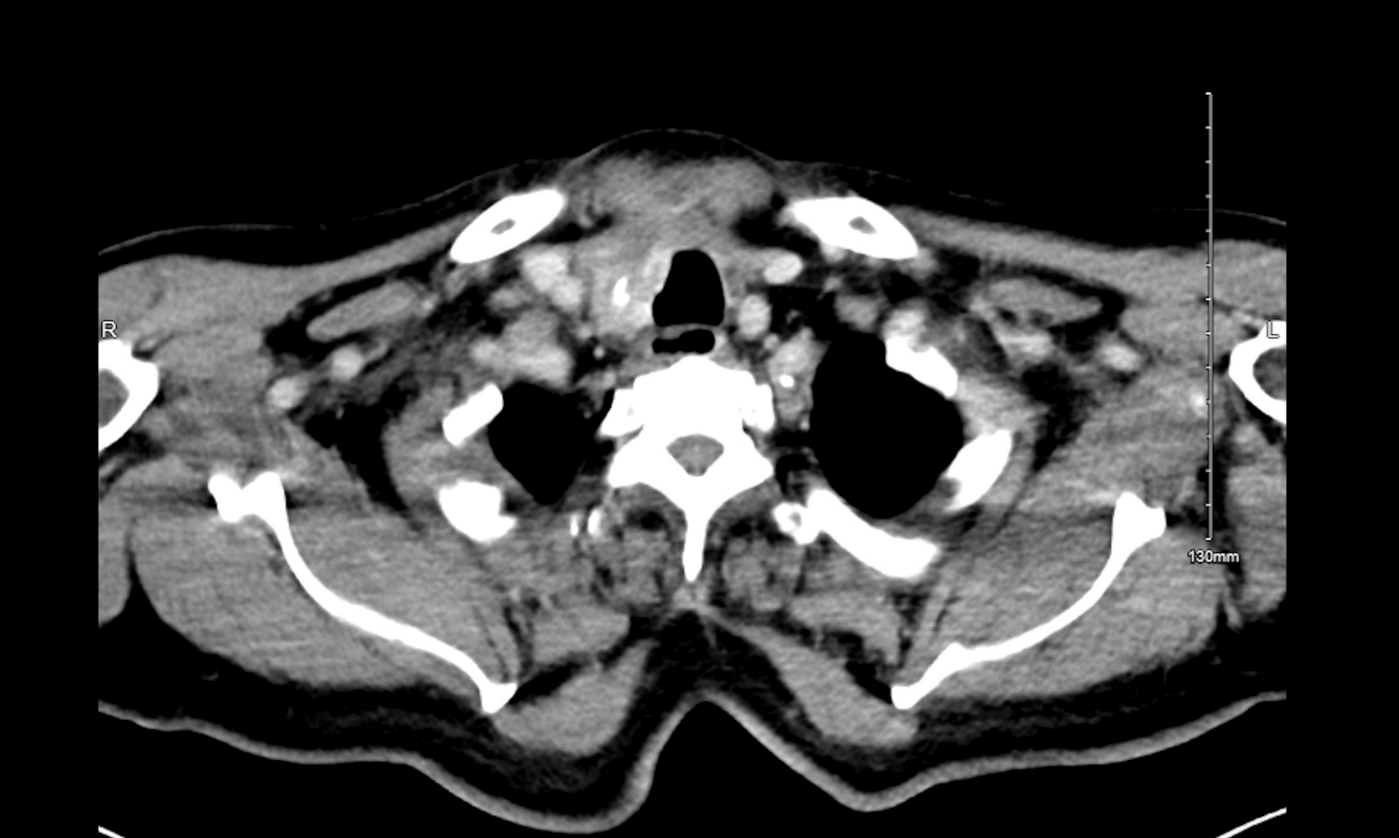
Staging CT scan. CT scan showing a cross-section of the neck with stable findings and no further metastasis.

Four months after the TTx, the patient underwent therapeutic RAI with a 131-whole body scan at a dose of 150 mCi that also showed intense uptake in the right thyroid bed and represented residual thyroid disease (Figure 3). Three months later, the patient came for a follow-up. Except for the hoarseness of voice, the patient was feeling well clinically. Further, the laboratory findings were as follows: calcitonin 2 pg/mL, CEA 4.3 ng/mL, TG 0.5 ng/mL, thyroglobulin antibody 9.8 ng/mL, and TSH 0.0 mIU/L. The final staging CT scan showed no further progression or metastasis (Figure 4).

## Discussion

The discovery of incidental thyroid carcinomas occurs relatively frequently, particularly with the increased use of advanced imaging techniques. The prevalence varies, but studies have shown that incidental thyroid nodules are found in 19% to 68% of patients who undergo imaging for non-thyroid-related reasons, such as CT scans, MRIs, and US performed for other conditions. Upon further investigation, approximately 5% to 15% of these incidental thyroid nodules are malignant [5-7].

Historically, PTC is the most common thyroid malignancy that accounts for more than 70%-85% of thyroid carcinomas and is derived from TG-producing follicular epithelial cells [1-3]. Conversely, MTC is a rare thyroid malignancy that accounts for only 5%-8% of thyroid malignancies and is a well-differentiated neuroendocrine malignancy that originates from the parafollicular calcitonin-producing cells, also known as C-cells [6-8]. The incidence of the two forms of carcinomas varies, and over the past two decades, the incidence of MTC‐PTC has increased from 2.7% to 12.3% of all MTCs [3]. The concomitant presence of PTC and MTC is rare, and presentation patterns are classified typically into the following two types: mixed MTC and PTC within the same neoplastic nodule or separate nodules within the thyroid gland.

The patient’s initial condition consisted of a non-displaced lift, second, third, and fourth rib fractures, and a sternal hematoma; however, the bilateral thyroid nodules with an invasion of the right side of the trachea were discovered incidentally only after the initial CT. During our assessment, the US showed five thyroid nodules, three right nodules of TIRAD-4, and two left thyroid nodules of TIRAD-3. Immediately thereafter, an FNA was performed on the most prominent nodules on both sides; however, the results were inconclusive. Usually, a preoperative diagnosis of thyroid carcinomas is secured based on thyroid FNA results. However, similar to our setting, indeterminate nodules may occur, including suspected follicular neoplasms and atypical cells of uncertain significance. Approximately 1%-2% of such cases are found to be MTC only after thyroidectomy [9]. Another study showed that 2.8% of MTC patients had concurrent PTC or FTC on surgical pathology, although many of the differentiated thyroid carcinomas (DTCs) were microcarcinomas discovered incidentally in the setting of known MTC [10]. A higher prevalence of mixed MTC and PTC in thyroidectomy specimens was seen in a retrospective study of 196 consecutive cases of MTC; of these, 27 (13.8%) were found to have coincidental microscopic PTC [11]. A smaller study by Kim et al. [12] found a 19% prevalence of coincidental PTC in their MTC series.

As aforementioned, the initial treatment and management of thyroid nodules usually begin by evaluation with an FNA biopsy, which is essential to diagnose thyroid pathology, particularly for nodules 1 cm or larger, depending upon US characteristics. Inconclusive FNA findings suggest MTC and should prompt further investigation, including calcitonin measurement and immunohistochemistry staining for such markers as calcitonin, chromogranin, and CEA. Comprehensive evaluations, including genetic testing for *RET* mutations, US, and contrast-enhanced imaging, are recommended for patients diagnosed with MTC. In the absence of metastases, TTx with central lymph node dissection is advised, with possible lateral node dissection based on serum calcitonin levels. Postoperative management includes monitoring serum calcitonin and CEA levels regularly with imaging follow-up if levels exceed 150 pg/mL [13].

During our patient’s TTx, the surgeon noticed an invasion of the right lower thyroid lobe of the trachea. However, as there was no tissue diagnosis and staging at that time, the surgeon decided not to remove the tracheal lesion. Tracheal lesions associated with thyroid nodules or carcinomas are relatively rare, particularly in more advanced or aggressive forms of thyroid cancer. In one study, approximately 10%-15% of patients with locally advanced thyroid cancer had invasion into the trachea and surrounding structures. According to another report, tracheal invasion occurs in approximately 7%-8% of differentiated thyroid cancers that present as locally advanced diseases [14-18]. The frequency and nature of tracheal involvement can vary based on the type and stage of thyroid carcinoma. Tracheal invasion is seen more commonly in advanced thyroid carcinomas, particularly ATC, and sometimes in aggressive forms of PTC and FTC. MTC can also invade the trachea, which is less common than in other aggressive types [3].

Upon further assessment, the patient’s gross extrathyroidal extension, incomplete resection, and laboratory levels showed that the patient had a high risk of PTC with invasion of the trachea (Figure 1). We recommended a revision of TTx, but the patient refused further surgery. Instead, the patient received diagnostic RAI with a 131-whole body scan at a dose of 150 mCi, which resulted in intense uptake in the proper thyroid tissue. A prospective multicenter study on high-risk ATA patients demonstrated a significant improvement in both disease-specific and overall mortality, as well as disease-free survival, in those with NTCTCSG stage III and IV thyroid cancer [19]. This finding was based on statistical adjustments using multivariate and propensity-stratified analyses. In addition, data collected prospectively from the Surveillance, Epidemiology, and End Results cancer registry indicated that postsurgical RAI therapy is associated with improved survival overall in patients with PTC who have distant metastases, particularly when these metastases are coupled with factors such as age over 45 years, tumor size that exceeds 2 cm, and positive lymph nodes at the time of primary diagnosis [20]. Further, rather than surgical resection, this approach may be helpful for patients when completing their thyroidectomy carries some increased risk and when they find a delay in the length of time required to ablate the normal thyroid acceptable. Thus, routine postsurgical RAI treatment is recommended in patients with ATA high-risk DTC.

## Conclusions

We present an incidental finding of concomitant MTC-PTC in the right lobe and PTC in the left lobe of the thyroid, which were first inconclusive by FNA, but had PTC invasion on the right side of the trachea during TTx. There has been a noted increase in concomitant MTC-PTC prevalence, from 2.7 to 12% in all MTCs. Moreover, this report contributes to the literature, particularly by highlighting the importance of raising awareness of the possibility of multiple types of carcinomas in a single thyroid gland, as presented in our case, because this rare phenomenon illustrates the complexity and heterogeneity of thyroid malignancy.
